# Efficient hybrid 3D system calibration for magnetic particle imaging systems using a dedicated device

**DOI:** 10.1038/s41598-020-75122-5

**Published:** 2020-10-28

**Authors:** Anselm von Gladiss, Matthias Graeser, André Behrends, Xin Chen, Thorsten M. Buzug

**Affiliations:** 1 Institute of Medical Engineering, University of Lübeck, 23562 Lübeck, Germany; 2 Section for Biomedical Imaging, University Medical Center Hamburg-Eppendorf, 22529 Hamburg, Germany; 3 Institute for Biomedical Imaging, Hamburg University of Technology, 21073 Hamburg, Germany; 4 Fraunhofer Research Institution for Individualized and Cell-Based Medical Engineering, 23562 Lübeck, Germany

**Keywords:** Medical imaging, Three-dimensional imaging

## Abstract

Image reconstruction in magnetic particle imaging is often performed using a system matrix based approach. The acquisition of a system matrix is a time-consuming calibration which may take several weeks and thus, is not feasible for a clinical device. Due to hardware characteristics of the receive chain, a system matrix may not even be used in similar devices but has to be acquired for each imager. In this work, a dedicated device is used for measuring a hybrid system matrix. It is shown that the measurement time of a 3D system matrix is reduced by 96%. The transfer function of the receive chains is measured, which allows the use of the same system matrix in multiple devices. Equivalent image reconstruction results are reached using the hybrid system matrix. Furthermore, the inhomogeneous sensitivity profile of receive coils is successfully applied to a hybrid system matrix. It is shown that each aspect of signal acquisition in magnetic particle imaging can be taken into account using hybrid system matrices. It is favourable to use a hybrid system matrix for image reconstruction in terms of measurement time, signal-to-noise ratio and discretisation.

## Introduction

Magnetic Particle Imaging (MPI) is a fast developing imaging modality, which lately entered the clinical scale^1, 2^. It depends on the nonlinear magnetisation behaviour of magnetic nanoparticles, which are used as tracer^3^. Due to the two coupled relaxation mechanisms, the Neel and Brown rotation, the magnetisation response of the nanoparticles can differ strongly for different particle properties^4^, different environments like binding state or viscosity and different particle shells^5^. While this can affect the ability to quantify the data, it can also be exploited to determine the environment of the nanoparticles on a molecular level. Depending on all these parameters the nanoparticles align in an external magnetic field, causing higher harmonic signals within the received signal. To reconstruct an image from this data two different approaches are performed, the x-space reconstruction^6^ and the frequency space reconstruction^3^. While in the x-space reconstruction no prior knowledge of the particle sample is required to reconstruct a native image, it gains resolution by deconvolution with the multi dimensional point spread function of the tracer used. Thus, a short calibration measurement is necessary for best performance in the reconstruction process. The frequency based reconstruction requires a large dataset of calibration measurements. The signal response of a cubical sample is measured within the field-of-view (FOV) of an imaging unit. This signal response is then stored in a row of a system matrix. To reconstruct an image a weighted iterative solving scheme is performed to solve the equation $\mathbf{S}\cdot \mathbf{c}=\mathbf{u}$ with $\mathbf{S}$ the system matrix, $\mathbf{u}$ the spectrum of the measured voltage and $\mathbf{c}$ the unknown spatial particle distribution. Although the reconstruction showed better results for complex encoding schemes like Lissajous schemes, it has some major drawbacks compared to the x-space approach. First, it is a time consuming process as it has to measure a large dataset of positions to achieve a good image discretisation. This discretisation should be considerably below the physical resolution of the system. In recent work, a resolution of 5 mm was achieved with a gradient strength of ${0.25}\,\,{\text{T m}}^{-1}$. This suggests that preclinical systems with ${2.5}\,{\text{T m}}^{-1}$ should provide a resolution of ${500}\,{\upmu \text{m}}$. However, this does not hold for systems using the frequency space approach. One reason is, that system matrices are normally not recorded using a grid of below 1 mm. This leads to an aliasing effect of the frequency patterns. However, due to the drop in signal-to-noise ratio (SNR) which scales with volume, the weak signals which would provide the improved resolution gets lost in the systems noise. As the SNR drops linearly with the sample volume, grids below 1 mm become challenging. One solution for this is to scale down the gradient for the system calibration and move the sample over the larger FOV using the same voxel spacing^7^. For a sufficient linear gradient this enables to reconstruct measured data of a higher gradient without introduction of artefacts or image distortions. However, in a real system this cannot be driven far as field distortions are present in the vicinity of the field generating coils^8^. In addition, the increase in voxel count leads to even longer calibration times which blocks the imaging system for clinical use.

An alternative for recording the system matrix is to use the so called hybrid system calibration by a magnetic particle spectrometer (MPS) with programmable offset fields^9–11^. Here, the oscillating drive fields are superimposed by homogeneous offset fields. By this, the whole field sequence resembles the sequence in an infinite small point within the scanner, thus removing the coupling of sample size and voxel size. The signal response of the tracer is then recorded by the system. In addition, the field generator can be build in a way that the offset fields can be swept fast between static values, enabling the measurement of up to 20 voxels per second. Due to the small bore size, the SNR is much higher resulting in a high quality system matrix with low voxel spacing in low measurement time.

In this work, we present three major advancements to the calibration scheme using magnetic particle spectroscopy. First, a three dimensional MPS system^12^ is extended to enable the superposition of a three dimensional Lissajous trajectory with a three dimensional offset field. System matrices are measured both with this MPS and a commercially available preclinical MPI scanner (MPI System 25/20FF, Bruker BioSpin, Ettlingen, Germany). Second, the transfer functions of the different analog receive chains are measured and a correction is successfully applied to the measured system matrices. A renal phantom which has been measured in the Bruker scanner is reconstructed using both system matrices. And third, it will be shown that the sensitivity profile of a receive coil can be applied successfully on a hybrid system matrix in order to avoid imaging artefacts and obtain quantitative results.

## Material and methods

### 3D MPS

The three-dimensional coil setup presented in^12^ has been extended to enable the superposition of a three-dimensional magnetic offset field to the magnetic excitation field. The magnetic offset fields are generated by mono-polar direct current sources (Delta Electronica, SM-800) which allow for an analog input and therefore, a very fast programming^11^. The polarities of the direct current sources are altered after acquiring one whole octant of the hybrid system matrix.

**Figure 1:**
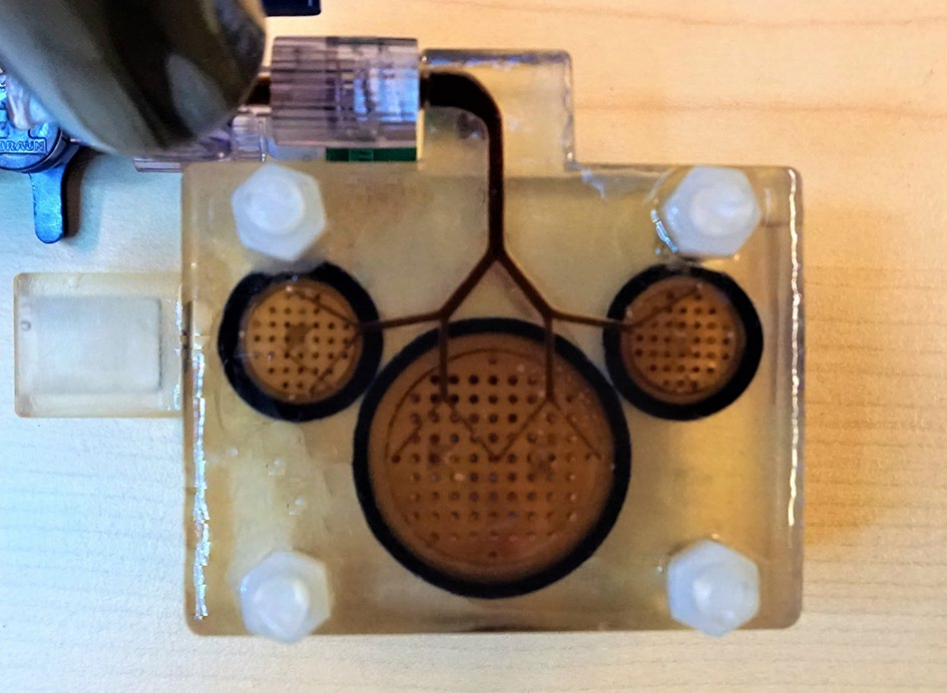
Top-view of a renal phantom for MPI measurements. It consists of three cylinders that are connected both at the top and bottom to a capillary structure. The cylinders feature a height of 10 mm and diameters of 20.5 mm and 10 mm, respectively. The diameter of the capillary structures varies from 0.7 mm to 2 mm. In total, the phantom features a height of 22 mm. The phantom has been presented first in^13^.

**Figure 2:**
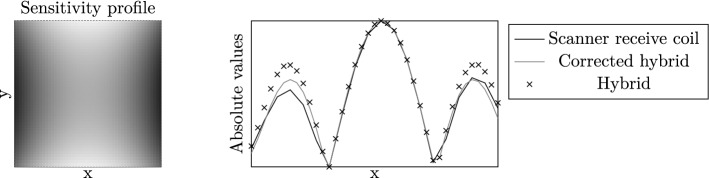
Correction of a hybrid system function with a sensitivity profile of a receive coil. The sensitivity profile of a gradiometric receive coil (left) has been simulated using Biot-Savart law. A system matrix has been measured using the receive coil. Normalised line profiles of the system function and a corresponding hybrid system function at the fifth harmonic of the excitation frequency are shown on the right. The system functions differ regarding the amplitudes of the side wave peaks. Correcting the hybrid system function with the simulated sensitivity profile, reduces the differences to the system function of the receive coil. However, the different amplitudes of the left and right side wave cannot be matched by the hybrid system function. They may be explained by a tilting of the receive coil which is not included in the simulated data.

**Figure 3:**
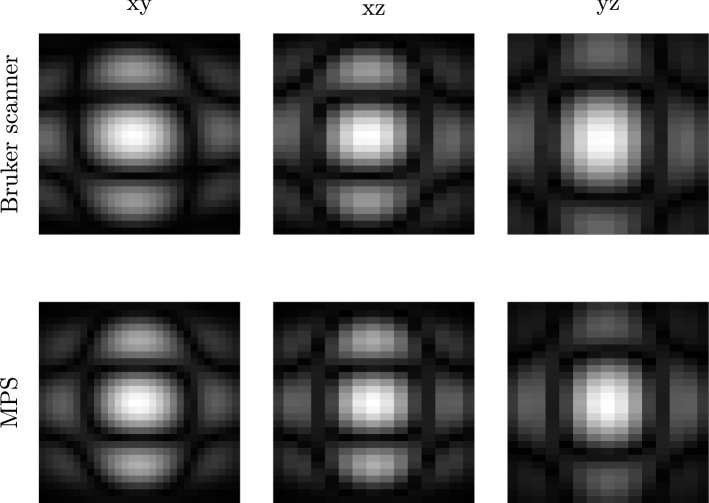
3D system functions at 176.12 kHz acquired with the Bruker scanner (top) and an MPS (bottom). The centre planes of xy, xz and yz are displayed. The system matrices have been acquired using a discretisation of ${29 \times 29 \times 15}$ voxels. Both the system matrices show the same structures with little difference at 176.12 kHz. The hybrid system matrix is symmetric. There are the connecting structures between the second wave peaks in all the four quadrants, whereas these structures are partly missing in the Bruker scanner’s system function. Furthermore, the system matrix of the Bruker scanner is shifted in the direction of z. In comparison to the hybrid system functions, it is shifted to the left in both the xz- and yz-plane.

**Figure 4:**
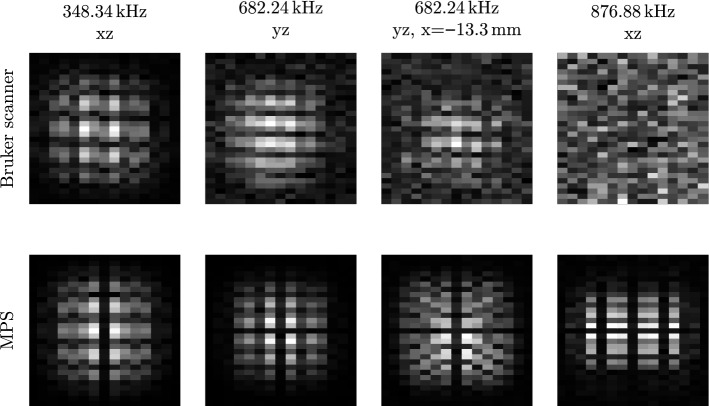
Noise influence on 3D system functions of higher frequency components that have been acquired with the Bruker scanner (top) and an MPS (bottom). The centre planes of the yz- and xz-plane are shown in the first, second and fourth column. The receive signal has been averaged 200 times for the Bruker scanner and 5 times for the MPS, respectively. The structures of the Bruker scanner’s system function at 348.34 kHz are blurry in comparison to the corresponding hybrid system function (first column). The central line (top to bottom) of the hybrid system function shows very small intensity values and thus, matches the zero amplitude value of the centre, which is given by e.g. Langevin theory. The Bruker scanner’s system function carries strong signal here, which may be caused by the size of the particle sample inside the Bruker scanner, that was bigger than the size of one voxel. Additionally, the particle sample may not have been centred accurately in the FOV of the Bruker scanner. This effect increases for the system function at 682.24 kHz (second column). Furthermore, it can be seen that the SNR of the hybrid system function is higher at 682.24 kHz, as the background of the Bruker scanner’s system function is noisy. In off-centre yz-planes (third column), the structures of the Bruker scanner’s system function can be barely seen, whereas the SNR of the hybrid system function still provides high SNR. For the centre planes of high frequency components (876.88 kHz, fourth column), the system function pattern cannot be identified anymore for the Bruker scanner. The corresponding hybrid system function still shows a good SNR.

**Figure 5:**
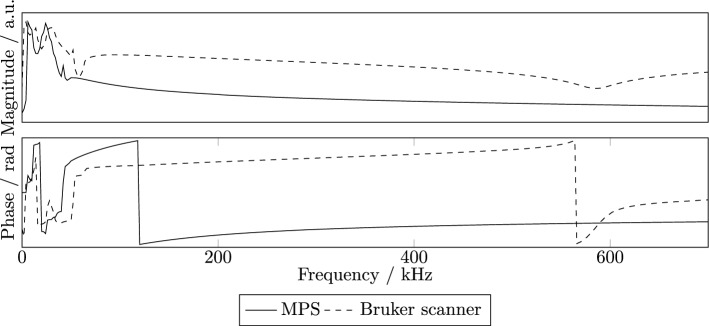
Measured transfer function seperated by amplitude and phase of the x-channel of the 3D MPS and the Bruker scanner.

**Figure 6:**
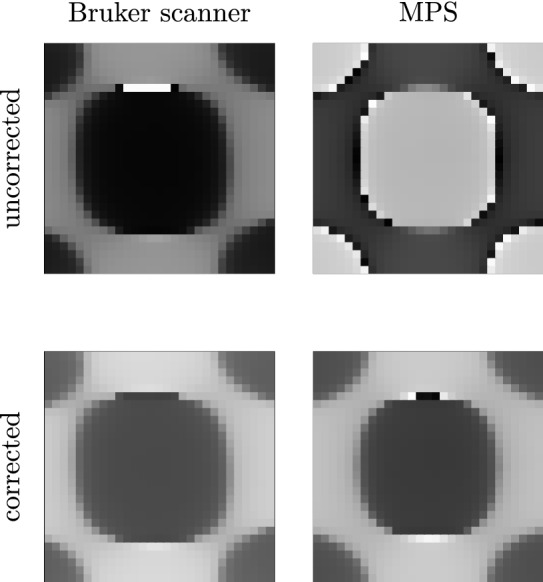
Phase visualisation of a system function measured at 127.10 kHz with the Bruker scanner (left) and an MPS (right). The images are windowed to the value range $\left[ -\pi , \pi \right]$. Before correcting the system functions with the transfer functions of the receive chains (top) the system functions show similar structures but have different phase values. After correcting the system functions (bottom), the phase values match better.

**Figure 7:**
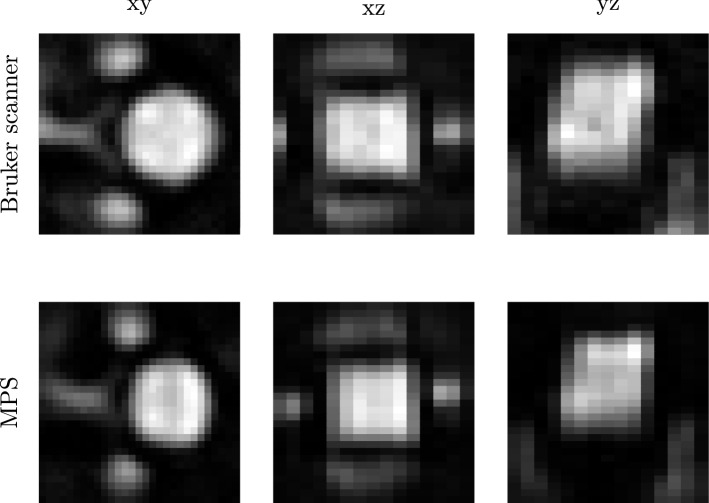
Reconstructed projection images of the measured renal phantom. The phantom has been reconstructed using a robot-based (top) and a hybrid system matrix (bottom). Both reconstruction sets show the same shape of the phantom. However, differences as a less detailed reconstruction of the capillary structures using the hybrid system matrix are visible. Furthermore, the reconstructed images using the robot-based system matrix are shifted in z-direction. This can be explained by the shifted system matrix as shown in Fig. 3.

**Figure 8:**
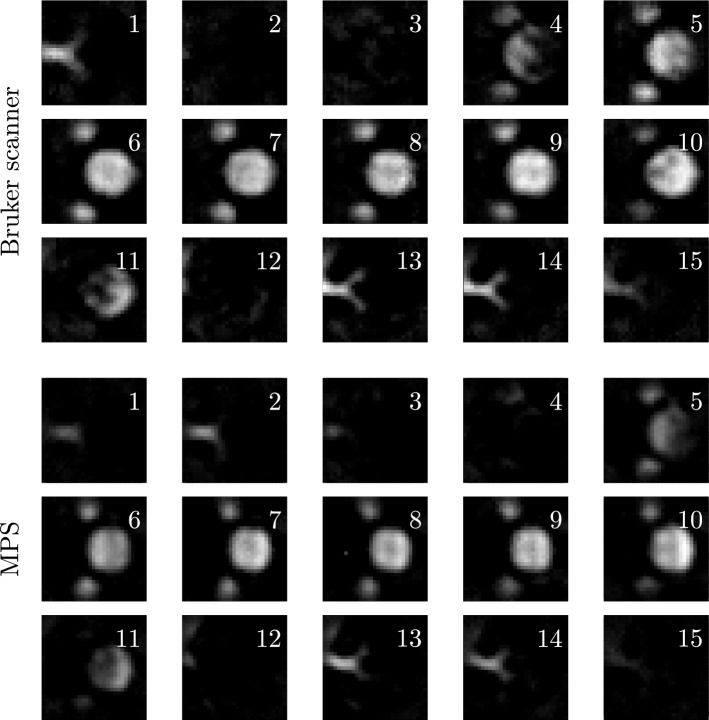
Reconstructed tomographic images of the measured renal phantom. The different slices in z-direction are shown for both the reconstructions using the robot-based (top) and the hybrid system matrix (bottom). The slices have been windowed with the minimum and maximum values of the reconstructed dataset. The three cylinders are reconstructed in the central slices (6–10). The large cylinder blurs at the right side when reconstructing with the system matrix measured with the Bruker scanner. In comparison, it is reconstructed with a sharp contour using the hybrid system matrix. The upper capillary structure (slices 13 and 14) are reconstructed very similar. The lower capillary structure can be identified only in the first slice using the Bruker scanner’s system matrix. It is reconstructed into the first two slices using the hybrid system matrix. This reconstruction difference may be explained by the shift of the robot-based system matrix in z-direction (see Fig. 3) and furthermore, indicates an inhomogeneity of the magnetic gradient field of the Bruker scanner.

**Figure 9:**
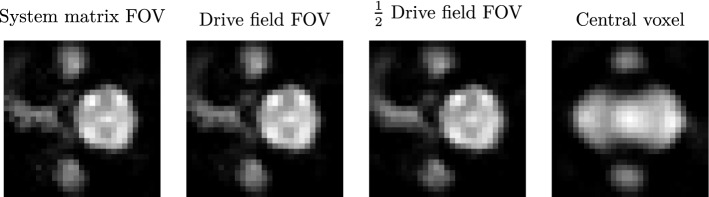
Reconstructed projection images of the renal phantom using a hybrid system matrix corrected with estimated transfer functions. For estimating the transfer function, different central segments of measured system matrices have been used. When using all the voxels available (corresponding to the system matrix FOV), the capillary structures are reconstructed only partially. There is background noise near to the small cylinders. Reconstruction of the capillary structures improves when using the voxels corresponding to the drive field FOV. When reducing the number of voxels to half of the drive field FOV, the background noise becomes lowest. Furthermore, the capillary structures are displayed fully. Reconstruction fails when using only the central voxel for estimating the transfer function.

**Figure 10:**
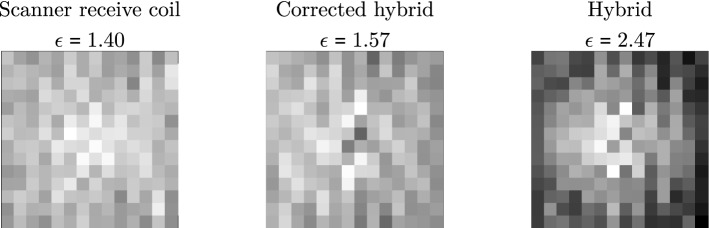
Comparison of the distribution of summed grey values for reconstructed images. A particle sample has been measured at ${14 \times 14}$  pixel positions. A gradiometric receive coil featuring an inhomogeneous sensitivity profile (see Fig. 2) has been used. The particle-position measurements have been reconstructed first using a system matrix measured with the receive coil, second using a hybrid system matrix and third, using a hybrid system matrix corrected by the sensitivity profile. The images show the summed grey values of the ${14 \times 14}$  reconstructed images mapped to the sample position. The ratio between maximum and minimum value of the grey scale is constant. Using the uncorrected hybrid system matrix (right), the reconstructed images of the particle-position measurement are brighter if the particle sample is near to the centre of the FOV corresponding to the signal weighting of the sensitivity profile. The ratio $\epsilon$ of maximum and minimum summed grey value is 1.76 higher as when reconstructing the system matrix measured with the receive coil (left). Here, the sensitivity profile is encoded in the system matrix leading to a more homogeneous distribution of summed grey values. After correcting the hybrid system matrix using the sensitivity profile (centre), the ratio value $\epsilon$ is comparable to when using the system matrix of the receive coil. The spatial weighting at the centre of the FOV cannot be observed anymore.

**Figure 11:**
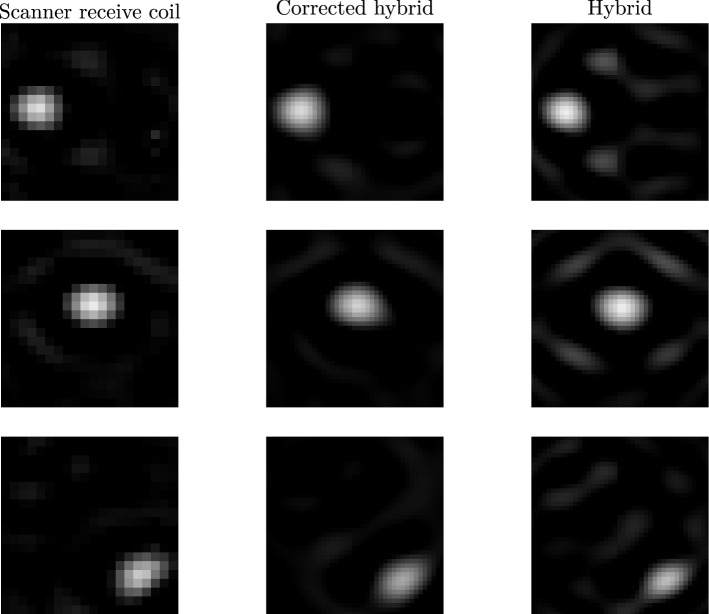
Comparison of reconstructed images of a particle-position measurement using a system matrix measured with a receive coil featuring an inhomogeneous sensitivity profile (left), using a hybrid system matrix (right) and using a hybrid system matrix corrected by the sensitivity profile (centre). The images have been reconstructed using an unregularised Kaczmarz-algorithm featuring one iteration and a constant frequency component selection. Using the hybrid system matrix without sensitivity correction lead to strong artefacts in the reconstructed images (right). Most of these artefacts cannot be identified when reconstructing with the corrected hybrid system matrix (centre). The remaining artefacts, however, are visible as well in the reconstructed images using the system matrix of the Bruker receive coil (left).

### System matrix

A robot-based system matrix has been acquired with the Bruker scanner using three dimensional excitation. The amplitudes of the excitation field have been 12 mT in each direction. The strength of the gradient field has been set to ${1.2}\,{\text{T m}}^{-1}$ in z-direction and ${0.6}\,{\text{T m}}^{-1}$ in x- and y-direction, respectively. Therefore, the drive field FOV size was ${40 \times 40 \times 20}$ mm. The FOV of the system matrix was set to ${46.67 \times 46.67 \times 23.33}$ mm and has been discretised into ${29 \times 29 \times 15}$ voxels. Hence, an overscan has been included in order to reduce reconstruction artefacts that may arise because of particles outside the drive field FOV^14^. A particle sample of Perimag (micromod Partikeltechnologie GmbH, Rostock; ${4}\, {\upmu \text{l}}, {0.151}\, \text{mmol}_{\mathrm{Fe}}\,\text{ml}^{1}$, ${8.5}\, \text{mg}_{\mathrm{Fe}}\,\text{ml}^{-1}$) has been used^15^. The receive signal has been averaged 200 times.

A hybrid system matrix has been measured with the mentioned 3D MPS. The excitation field amplitudes were set to the same value as in the Bruker scanner (12 mT). The FOV of the Bruker scanner’s system matrix was ${46.67}\,\mathrm{mm} \cdot {0.6}\,\mathrm{T m}^{-1} = {28} \,\mathrm{mT}$ which corresponds to $\left[ {-14}\,\mathrm{mT}, {14}\,\mathrm{mT}\right]$ in both x- and y-direction and ${23.33}\,\mathrm{mm}\cdot {1.2}\,{\text{T m}^{-1}} = {28}\,\mathrm{mT}$ which corresponds to $\left[ {-14}\,\mathrm{mT},{14}\,\mathrm{mT}\right]$ in z-direction, respectively. To match the same field sequences the magnetic offset fields inside the MPS were varied in the range of $\left[ {-14}\,\mathrm{mT},{14}\,\mathrm{mT}\right]$ in steps of 1 mT in x- and y-direction and 2 mT in z-direction, which results in a hybrid system matrix of ${29\times 29\times 15}$  voxels. A particle sample of Perimag (${60} \,\upmu \mathrm{l}, {12.5}\, {\mathrm{mg}/\mathrm{ml}}$) has been used. The receive signal has been averaged 5 times.

### Transfer function

Given constant measurement parameters and a constant particle sample, the receive signals of two systems (e.g. an MPI scanner and an MPS) differ due to the transfer function of the receive chain. The transfer function encodes the signal attenuation and phase shift which are introduced by e.g. amplifiers and filters. In^11, 16^ the transfer function has been estimated for reconstructing measured data using a hybrid system matrix. Here, the transfer functions of the receive chains are measured.

The transfer function of the receive paths of both the Bruker scanner and the MPS are determined by the ratio between the receive signal and a known magnetic moment *m* inside the FOV and the measurement chamber, respectively. An alternating current driven through a calibration coil that is sensitive parallel to the receive coil generates the magnetic moment *m*

1 $$\begin{aligned} m(t) = I(t) \cdot N \cdot A = \frac{U^m(t)}{R} \cdot N \cdot A. \end{aligned}$$

Here, *A* and *N* are the cross section and the number of turns of the calibration coil, respectively. The current *I*(*t*) is determined by the voltage $U^m(t)$, which is the voltage corresponding to the generated magnetic moment and is measured using a known serial resistance *R*.

The frequency components of the transfer function $a_k$ are computed by the ratio of the frequency components of the receive signal $u_k$ and the magnetic moment $m_k$

2 $$\begin{aligned} a_k = \frac{u_k}{m_k} = \frac{u_k \cdot R}{u^m_k \cdot A\cdot N} . \end{aligned}$$

The dimension of a transfer function of a receive path is $\left[ {\frac{\mathrm{v}}{\mathrm{A m}^2 }} \right]$. Applying the transfer function to a receive signal results in the dimension $\left[ {\text{A m}^{2}} \right]$ of the receive signal, which is the dimension of the magnetic moment.

For comparison, a transfer function is estimated as described in^11^. In order to analyse the influence of the chosen voxels for estimation, four centred segments featuring ${29\times 29\times 15}$, ${25\times 25\times 13}$, ${13\times 13\times 7}$ and ${1\times 1\times 1}$  voxels are selected. The four segments correspond to the system matrix FOV including an overscan, the drive field FOV, half of the drive field FOV and the central voxel solely.

### Phantom data

A renal phantom is shown in Fig. 1, which has been designed for MPI measurements^13^. It consists of three cylinders that are connected to capillary structures both on the top and bottom. The phantom has been filled with Perimag featuring a concentration of 0.236 mg/ml and has been measured in the Bruker scanner using a gradient field strength of ${1.2} \,{\text{Tm}^{-1}}$ in z-direction and ${0.6}\,{\text{Tm}}^{-1}$ in x- and y-direction. The receive signal has been averaged 1000 times to ensure a high SNR.

In order to reconstruct the phantom data with the hybrid system matrix, both the hybrid system matrix and phantom data are corrected for the transfer functions of the receive paths. For comparison, the phantom data are also reconstructed using the hybrid system matrix which has been corrected with estimated transfer functions.

### Sensitivity profile

The sensitivity profile of the receive coil is a spatial weighting of the particle concentration^17^. In case of image reconstruction using a hybrid system matrix this spatial weighting is different as the MPS has no spatial dependent receive profile. Uncorrected, this would lead to a highlighting in regions where the receive coil of the MPI scanner has a high sensitivity e. g. near to the turns. The transfer function corrects the relative sensitivity of both systems already for the centre point. However, the spatial weighting of the receive coil is not corrected. Therefore, a sensitivity profile, normalised to the coil centre has to be determined. This can either be done by simulating the coils sensitivity profile using Biot-Savart law, or by measuring the spatial dependence of the transfer function on the system matrix grid. The resulting sensitivity profile can then be multiplied with the transfer function corrected system matrix and can be used for reconstruction.

In order to demonstrate the sensitivity profile correction, an experiment using a gradiometric receive coil featuring an inhomogeneous sensitivity profile was performed. A particle sample was moved to ${14\times 14}$  positions of the FOV and measured. The measurements were reconstructed using a hybrid system matrix and a system matrix acquired using the receive coil. The grey values of the reconstructed images were summed and the sum was mapped back to the corresponding spatial position of the particle sample for comparison. In detail:

A gradiometric receive coil has been installed inside the Bruker scanner and used instead of the native receive channel x. A 2D system matrix has been measured with the Bruker scanner featuring drive field strengths of 12 mT and gradient field strengths of ${1}\,{\text{T m}}^{-1}$ in both x- and y-direction resulting in a drive field FOV of ${24\,\text{mm} \times 24}\,{\text{mm}}$. However, only a subset of $20 \,\text{mm} \times 20\,\text{mm}$ has been measured using ${20\times 20}$ pixels. A particle sample of Perimag $({1}\,{\upmu \mathrm{l}}, {25} \,{\text{mg}/\text{ml}})$ has been used. The receive signal has been averaged 100 times.

A second, smaller system matrix has been measured using the same particle sample and a discretisation of ${14\times 14}$ pixels corresponding to a system matrix FOV of ${14\,\text{mm}\times 14 \,\text{mm}}$. The measurement parameters have been the same as for the first system matrix. This second system matrix will be used for reconstructing ${14\,\text{mm}\times 14\, \text{mm}}$ particle-position measurements.

A hybrid 2D system matrix has been measured using the same drive field strengths. The magnetic offset fields have been varied using a step width of 0.5 mT in the range of $\left[ {-14}\,\mathrm{mT}, {14}\,\mathrm{mT}\right]$ in both x- and y-direction resulting in a grid size of ${57\times 57}$  pixels corresponding to a FOV of ${28\,\mathrm{mm} \times 28}\,\mathrm{mm}$ inside the Bruker scanner. A particle sample of Perimag $({60}\,{\upmu \mathrm{l}}, {12.5} \,{\mathrm{mg}/\mathrm{ml}})$ has been used. The receive signal has been averaged 50 times.

The sensitivity profile of the gradiometric receive coil has been simulated using Biot-Savart law (see Fig. 2 left). The hybrid system matrix has been corrected for sensitivity profile by a pointwise multiplication.

The ${14\times 14}$  particle-position measurements have been reconstructed using first the system matrix of the Bruker scanner, second the uncorrected hybrid system matrix and third, the corrected hybrid system matrix. The frequency components selected have been the same for all reconstructions. An unregularised Kaczmarz algorithm using one iteration has been used.

## Results

### System matrix

The absolute value of system functions at 176.12 kHz is shown in Fig. 3. The system functions have been acquired both in the Bruker scanner and an MPS. The centre planes in each spatial direction are shown. The hybrid system matrix reveals connecting structures between the second wave peaks (counted from the centre) for each centre plane, whereas these structures cannot be identified everywhere in the Bruker scanner’s system matrix. There is a shift in z-direction (left) for the xz- and yz-plane for the system matrix of the Bruker scanner. The corresponding planes of the hybrid system matrix are symmetric.

Figure 4 shows that the SNR of the hybrid system matrix is very high in comparison to the Bruker scanner’s system matrix. The calculated SNR values for the three system functions shown at 348.34 kHz, 682.24 kHz and 876.88 kHz are 1727, 127 and 57 for the hybrid system functions and 28, 3 and 1 for the Bruker scanner’s system functions. Outside the centre plane (third column) the structures of the system function measured with the Bruker scanner cannot be identified at 682.24 kHz. At high frequency components (876.88 kHz, fourth column) the structures are not visible anymore due to noise. The structures of the hybrid system matrix feature a high SNR in both cases. Furthermore, the central plane of the hybrid system matrix shows very low intensity values in x- and y-direction (top to bottom) at 348.34 kHz and 682.24 kHz (first and second column), respectively, whereas this separating structure is blurred for the Bruker scanner’s system matrix. Zero amplitude values are predicted here by e.g. Langevin theory for symmetric excitation. However, in a real measurement there is slight signal contribution present as in the hybrid system matrix due to small magnetic offset fields such as the earth magnetic field.

The $29\cdot 29\cdot 15=12{,}615$ voxels of the system matrix have been measured within 20 h and 6 min with the Bruker scanner. As a background measurement has been taken after each 29 voxels, the total number of measurements was 13,054. Therefore, the measurement time for one sample position was 5.54 s on average. Due to the 200 averages of the receive signal, the measurement itself took 4.31 s while the robot movement time varied for different positions resulting in an average robot movement time of 1.23 s. Inside the MPS, the 12,615 voxels of the hybrid system matrix were measured in 53 min. 200 additional background measurements were taken in less than 30 s. The average measurement time is 0.25 s for a single voxel and background frame, respectively. The average measurement time consists of the actual measurement (0.11 s for 5 averages of the receive signal), direct current switching for varying the magnetic offset fields (0.02 s), initialisation of the MPS and waiting time when switching between different octants of the hybrid system matrix. These dead times (initialisation and waiting time) result in an additional measurement time of 0.12 s for one voxel. In total, the average measurement time per voxel is reduced by 96% when acquiring a hybrid system matrix.

### Transfer function

The system matrices measured with the Bruker scanner and the MPS hold the measured voltage values which are not comparable directly between the devices. A transfer function correction is performed for reconstructing measured phantom data with the hybrid system matrix and for translating the system matrices to a common base.

The measured transfer functions of the receive chain x of both the Bruker scanner and the MPS are shown in Fig. 5. In comparison to the estimated transfer function shown in^11^, the frequency components of the measured transfer functions show a high SNR above 50 kHz, where the transmission filter does not attenuate.

A system function at 127.10 kHz of the Bruker scanner and a hybrid one are both corrected for the corresponding measured transfer function (see Fig. 6). Before correction (top), the system functions show similar structures but the actual phase values differ. After the correction step (bottom), the phase values are similar.

### Phantom data

The system matrices and the measured renal phantom data have been corrected for the transfer functions of the receive chains and have now the dimension of the magnetic moment. Thus, the hybrid system matrices can be used for reconstructing the renal phantom.

The reconstruction results of the renal phantom are shown in Fig. 7. As a first overview, the whole phantom is shown as projection images in xy, xz and yz directions. Both the reconstructions using the system matrix of the Bruker scanner (top) and the hybrid one (bottom) show the same shapes of the renal phantom. However, the reconstruction results using the Bruker scanner’s system matrix are shifted in z-direction caused by the shift of the system functions shown in Fig. 3.

The single slices in z-direction are shown in Fig. 8. One capillary structure can be identified in the lower z-slices. Using the hybrid system matrix it is reconstructed into the slices 1 and 2, whereas it is only visible in the first slice for the Bruker scanner’s system matrix. Slices 6 to 10 show the three cylinders of the renal phantom. The contour of especially the big cylinder is reconstructed sharply using the hybrid system matrix. With the system matrix of the Bruker scanner, the contour blurs on the right side. The second capillary structure is being reconstructed mainly into the slices 13 and 14 for both system matrices and in more detail with the Bruker scanner’s system matrix.

For comparison, the renal phantom has also been reconstructed using the hybrid system matrix that has been corrected with estimated transfer functions. The reconstruction results are shown in Fig. 9 as projection images. When the transfer function is estimated using all the voxels available (system matrix FOV), the capillary structures are being reconstructed partially. Additionally, the background noise is high near to the small cylinders. The capillary structures are being reconstructed better when using only the voxels corresponding to the drive field FOV. The best reconstruction result can be achieved using the voxels inside half of the drive field FOV. Here, background noise is lowest and the capillary structures are reconstructed completely. The high SNR of the spectra in the centre of the FOV produce a better TF estimation than spectra of the outer voxels. Image reconstruction fails when using only the central voxel for estimating the transfer function. Overall, better image reconstruction results have been achieved using the measured transfer function compared to the estimated transfer functions.

### Sensitivity profile

In case of an inhomogeneous sensitivity profile of a receive coil, a hybrid system matrix has to be corrected for this as well for artefact-free image reconstruction.

The simulated sensitivity profile of the gradiometric receive coil is shown in Fig. 2 (left). Normalised line profiles of the fifth harmonic of the excitation frequency were measured with first a receive coil, second a hybrid approach and third, a hybrid approach corrected for the sensitivity profile (right). The side wave peaks of the first line profile are smaller in comparison to the profile of the hybrid system function. Furthermore, these side wave peaks are of different amplitude which may be caused by a tilted installation of the receive coil. After correcting the hybrid system function with the simulated sensitivity profile, the line profiles show only minor differences. The amplitudes of the central and right wave peaks match.

The grey values of each of the ${14\times 14}$ reconstructed images, each representing one sample position, have been summed in order to evaluate the sensitivity profile correction. The summed grey values are mapped to the corresponding spatial position of the particle sample (see Fig. 10). Reconstructing with the system matrix measured with the gradiometric receive coil generates a random distribution of summed intensity values (left). The ratio between highest and lowest summed grey value is $\epsilon = 1.40$. Here, a value of $\epsilon =1$ would mean that every reconstructed image had the same energy which could be expected for an ideal system. When reconstructing the ${14\times 14}$  particle-position measurements with the uncorrected hybrid system matrix, the distribution of summed grey values is inhomogeneous (right). The summed grey values are largest when the particle sample was placed in the centre of the FOV. The ratio $\epsilon = 2.47$ is 1.76 times as high as when reconstructing with the system matrix measured in the receive coil. After correcting the hybrid system matrix with the sensitivity profile, the ratio reduces to $\epsilon = 1.57$ which is close to the original case. A correlation between high grey value sums and sample positions at the centre of the FOV cannot be observed.

Reconstructed images of particle-position measurements at three different spatial positions are shown in Fig. 11. As the magnetic field discretisation of the hybrid system has been higher than the corresponding spatial disretisation of the system matrix measured with the receive coil, the spatial resolution is higher in the reconstructed images in the central and right column. Imaging artefacts are strong when reconstructing using the uncorrected hybrid system matrix (right). When correcting the hybrid system matrix with the sensitivity profile, most of the imaging artefacts disappear. The remaining artefacts, however, can also be identified in the images reconstructed with the system matrix measured in the receive coil itself.

## Discussion

### System matrix

It has been demonstrated that hybrid system matrices feature a very high SNR. Even with a small number of receive signal averages the SNR may be higher than a corresponding robot-based system matrix measured with a high number of averages. This finding in^11^ has been confirmed. As the particle sample size and discretisation of the FOV are not related for a hybrid system matrix, the magnetic field discretisation of the hybrid system matrix could be increased. This is essential when the physical spatial resolution of a measurement, which increases with the SNR, is higher than the spatial grid of the system matrix. Recently, an emulated spatial resolution of few hundred ${\upmu \mathrm{m}}$ has been achieved in reconstructed images using hybrid phantoms and MPI technology^18^. The acquisition of a conventional system matrix featuring a corresponding spatial grid would be difficult as the sample size would be very small and thus, the SNR of the system matrix would decrease.

The average measurement time for one voxel could be reduced by 96% using the hybrid system matrix. The movement time of the robot is replaced by the switching time of the direct current sources which lasts milliseconds. Hence, the measurement time of a hybrid system matrix is influenced highly by the number of receive signal averages. Due to the proximity of the particle sample to the receive coils inside an MPS, the SNR is high even for a small number of averages which enables a very fast hybrid system matrix acquisition.

The magnetic gradient field of the MPI scanning device has been assumed to be linear. Therefore, a linear grid of magnetic offset field positions has been measured in the MPS. However, a hybrid system matrix can be measured at arbitrary magnetic offset field positions, which allows for measuring the gradient field in an imaging device first and then emulating it including its inhomogeneities in the MPS.

### Phantom data

The measured phantom data have been reconstructed using both a robot-based and a hybrid system matrix featuring same excitation field, system matrix FOV and disrectisation of the FOV and magnetic field, respectively. The data have been corrected with measured receive chain transfer functions. The reconstruction results are similar. The spatial allocation of the structures differs in the reconstructed images, as the robot-based system matrix features both a shift in z-direction and inhomogeneities of the magnetic gradient field, which are not included in the hybrid system matrix. However, the same structures are reconstructed with a high imaging quality.

A comparable reconstruction result has been achieved with estimating the receive chain transfer function. However, the reconstruction results differ with the selection of voxels for transfer function estimation. Especially when using only the central voxel, reconstruction fails (see Fig. 9). As the system matrix measured in the Bruker scanner features a shift in z-direction (see Fig. 3), the central voxels of both the system matrices do not match. Then, transfer function estimation fails. When selecting a larger segment of voxels, the influence of the z-shift on the transfer function estimation decreases.

### Transfer function

For estimating the receive chain transfer function successfully, the calibration measurements have to be identical in different systems. Especially a difference in the spatial position of the particle sample inside the FOV or magnetic field has a direct influence on the quality of the transfer function estimation.

When estimating a transfer function, the ratio of frequency components of particle signal is calculated. Therefore, estimation fails for frequency components that do not carry particle signal. As the particle signal is specific, the frequency components carrying signal may vary for different particles. Hence, a transfer function would have to be estimated for each type of particles leading to numerous calibration measurements.

Although image reconstruction of measured data may be successful using an estimated transfer function, it is favourable to measure the transfer functions of the receive chains. First, a measured transfer function can be used for correcting arbitrary signals measured with the system. Second, correction with a measured transfer function results in the magnetic moment that has been detected, which can be used for multiple applications such as visualisation of the magnetisation curve of particles.

### Sensitivity profile

The line profiles of the measured system matrices in a gradiometric receive coil and an MPS, respectively, converge after correcting the hybrid system matrix with the sensitivity profile of the receive coil (see Fig. 2). When simulating the sensitivity profile, the exact positioning of the receive coil inside the magnetic field must be known. Here, a tilting of the receive coil has not been included in the simulation leading to a difference in the side wave peaks.

The heterogeneous distribution of summed grey values can be reduced by correcting the hybrid system matrix with the simulated sensitivity profile. When reconstructing with an uncorrected hybrid system matrix (Fig. 10, right), the summed grey values are much higher for particle sample positions in the centre of the FOV. This spatial weighting at the centre of the FOV cannot be identified when reconstructing with a corrected hybrid system matrix (Fig. 10, centre). However, the original ratio ($\epsilon =1.40$) between maximum and minimum summed grey value cannot be restored and may be explained by a tilting of the receive coil and the resulting error in correcting the hybrid system matrix.

The reconstructed images using the corrected hybrid system matrix and the system matrix measured inside the receive coil show the same imaging artefacts. Correcting a hybrid system matrix with the sensitivity profile of a receive coil does not only correct for the spatial weighting of the particle sample in the reconstructed image, but improves image reconstruction in terms of artefacts as well (see Fig. 11).

## Conclusion

In this work, it has been shown that it is favourable to measure the transfer function of a receive chain in contrast to estimating it. A measured transfer function provides a high SNR for all frequency components and thus, allows for reconstructing arbitrary signals.

The presented work showed solutions for the three main issues regarding system matrix reconstruction technique. First, the usage of an MPS for system calibration reduces the time needed for the determination of the system matrix and does not occupy the MPI scanner systems schedule. Second, it decouples the SNR from the sample volume, thus provides high SNR and high discretisation in one system matrix. And third, due to the high discretisation, partial volume effects or aliasing artefacts for large sample volumes are avoided.

A hybrid system matrix that has been corrected for the measured transfer function of the receive chain represents the actual magnetic moment of the particle sample in the system matrix FOV for each imaging device with the same magnetic field sequence. It is the golden truth for the dynamic behaviour inside an imaging device.

Inhomogeneous sensitivity profiles of dedicated receive coils can be applied successfully on a hybrid system matrix. Then, the hybrid system matrix does not correspond to the actual magnetic moment anymore but matches the receive signal in the imaging device and can be used for image reconstruction. Here, the sensitivity profile of the receive coils within the Bruker scanner have not been applied to the hybrid 3D system matrix. As the receive coils of the Bruker scanner have a large distance to the system matrix FOV, their sensitivity profiles were assumed to be homogeneous. However, if a hybrid system matrix is not corrected for an inhomogeneous sensitivity profile, image reconstruction artefacts such as streaking artefacts and a spatial weighting of voxels can been observed. In this work, the inhomogeneous sensitivity profile of a gradiometric receive coil has been applied to a hybrid system matrix and thus, image reconstruction artefacts have been avoided.

To test the suitability of the hybrid approach, the receive signal chain in MPI has been dissected and applied to the measurement of a hybrid system matrix. Given the same magnetic field sequence, particle sample and presented correction steps, a hybrid system matrix can replace the system matrix measured in an imaging device completely.

The measurement of a hybrid system matrix can be further optimised by underlying the actual magnetic gradient field of an imaging device including inhomogeneities. The gradient field can either be measured fully or approximated using spherical harmonics^19^. Then, hybrid system matrices can be used efficiently as well for multi-patch reconstruction^8^.

The excitation field of an imaging device may be inhomogeneous as well^20^ leading to different drive field amplitudes in the magnetic field sequence. Furthermore, the field lines may follow non straight pathways within the FOV i.e. for single-sided devices^21^. However, the magnetic field strength and coupling can be changed intentionally in an MPS for hybrid system matrix measurements^22^. Simulated or measured drive field data featuring inhomogeneities may therefore be used for measuring a hybrid system matrix for arbitrary field generator setups.

Thus, system matrices for a wide range of scanner geometries can be emulated in an MPS for efficient system matrix acquisition.
